# Prevalence of trypanosomes, salivary gland hypertrophy virus and *Wolbachia* in wild populations of tsetse flies from West Africa

**DOI:** 10.1186/s12866-018-1287-4

**Published:** 2018-11-23

**Authors:** Gisele M. S. Ouedraogo, Güler Demirbas-Uzel, Jean-Baptiste Rayaisse, Geoffrey Gimonneau, Astan C. Traore, Antonios Avgoustinos, Andrew G. Parker, Issa Sidibe, Anicet G. Ouedraogo, Amadou Traore, Bale Bayala, Marc J. B. Vreysen, Kostas Bourtzis, Adly m. M. Abd-Alla

**Affiliations:** 1 Insect Pest Control Laboratory, Joint FAO/IAEA Programme of Nuclear Techniques in Food and Agriculture, International Atomic Energy Agency, P.O. Box 100, A-1400 Vienna, Austria; 2 Ecole National de l’Elevage et de la Santé Animale, 03 BP 7026, Ouagadougou 03, Burkina Faso; 3 Université Ouaga 1 Professeur Joseph Ki-Zerbo, BP 7021, Ouagadougou 01, Burkina Faso; 4 Centre International de Recherche-Développement sur l’Elevage en zone Subhumide (CIRDES), 01 BP 454, Bobo-Dioulasso 01, Burkina Faso; 5 CIRAD, UMR INTERTRYP, F-34398 Montpellier, France; 6 Pan African Tsetse and Trypanosomosis Eradication Campaign (PATTEC), Bamako, Mali; 7 Pan African Tsetse and Trypanosomosis Eradication Campaign (PATTEC), Projet de Création de Zones Libérées Durablement de Tsé-tsé et de Trypanosomoses (PCZLD), Bobo-Dioulasso, Burkina Faso; 8 Institut du Développement Rural, Université Polytechnique de Bobo-Dioulasso, Bobo-Dioulasso, Burkina Faso; 9 Institut de l’Environnement et des Recherches Agricoles (INERA), BP 8635 Ouagadougou 04, Burkina Faso

**Keywords:** *Glossina* spp., Trypanosoma spp., *Wolbachia* spp., SGHV, Microbe infection rate, Interactions

## Abstract

**Background:**

Tsetse flies are vectors of African trypanosomes, protozoan parasites that cause sleeping sickness (or human African trypanosomosis) in humans and nagana (or animal African trypanosomosis) in livestock. In addition to trypanosomes, four symbiotic bacteria *Wigglesworthia glossinidia*, *Sodalis glossinidius, Wolbachia, Spiroplasma* and one pathogen, the salivary gland hypertrophy virus (SGHV), have been reported in different tsetse species. We evaluated the prevalence and coinfection dynamics between *Wolbachia*, trypanosomes, and SGHV in four tsetse species (*Glossina palpalis gambiensis*, *G. tachinoides, G. morsitans submorsitans*, and *G. medicorum*) that were collected between 2008 and 2015 from 46 geographical locations in West Africa, i.e. Burkina Faso, Mali, Ghana, Guinea, and Senegal.

**Results:**

The results indicated an overall low prevalence of SGHV and *Wolbachia* and a high prevalence of trypanosomes in the sampled wild tsetse populations. The prevalence of all three infections varied among tsetse species and sample origin. The highest trypanosome prevalence was found in *Glossina tachinoides* (61.1%) from Ghana and in *Glossina palpalis gambiensis* (43.7%) from Senegal. The trypanosome prevalence in the four species from Burkina Faso was lower, i.e. 39.6% in *Glossina medicorum,* 18.08%; in *Glossina morsitans submorsitans,* 16.8%; in *Glossina tachinoides* and 10.5% in *Glossina palpalis gambiensis*. The trypanosome prevalence in *Glossina palpalis gambiensis* was lowest in Mali (6.9%) and Guinea (2.2%). The prevalence of SGHV and *Wolbachia* was very low irrespective of location or tsetse species with an average of 1.7% for SGHV and 1.0% for *Wolbachia*. In some cases, mixed infections with different trypanosome species were detected. The highest prevalence of coinfection was *Trypanosoma vivax* and other *Trypanosoma* species *(*9.5%) followed by coinfection of *T. congolense* with other trypanosomes (7.5%). The prevalence of coinfection of *T. vivax* and *T. congolense* was (1.0%) and no mixed infection of trypanosomes, SGHV and *Wolbachia* was detected.

**Conclusion:**

The results indicated a high rate of trypanosome infection in tsetse wild populations in West African countries but lower infection rate of both *Wolbachia* and SGHV. Double or triple mixed trypanosome infections were found. In addition, mixed trypanosome and SGHV infections existed however no mixed infections of trypanosome and/or SGHV with *Wolbachia* were found.

**Electronic supplementary material:**

The online version of this article (10.1186/s12866-018-1287-4) contains supplementary material, which is available to authorized users.

## Background

Tsetse flies (*Glossina* sp.) are obligate blood feeding insects that transmit protozoan parasites (*Trypanosoma* spp.), the etiological agents of African trypanosomosis that cause sleeping sickness or human African trypanosomosis, (HAT) and nagana or animal African trypanosomosis, (AAT) in livestock [1, 2]. Both diseases cause many direct and indirect losses, which represent a major obstacle for sustainable development in endemic countries [3].

Trypanosomosis is enzootic in an area covering ca. 10 million km^2^ in sub-Saharan Africa and is transmitted by different species of tsetse flies that vary in their vectorial capacity for the different *Trypanosoma* species [2]. In West Africa, HAT is caused by *Trypanosoma brucei gambiense*, that accounts for over 90% of the globally reported HAT cases [4] and is mainly transmitted by tsetse flies from the palpalis group (*Glossina tachinoides, G. palpalis gambiensis* and *G. p. palpalis*) [5]. The AAT causative agents (*Trypanosoma vivax, T. congolense, T. brucei brucei* and *T. evansi)* are transmitted by a broader range of tsetse fly species which include, in addition to the above-mentioned palpalis group, also flies from the morsitans group (*G. morsitans submorsitans* and *G. longipalpis*) [6, 7]. There are 11 different pathogenic trypanosomes that can be characterized by molecular methods using specific or common primers [6–8].

Due to the lack of effective vaccines and inexpensive drugs for HAT and also the development of resistance of the AAT parasites against available trypanocidal drugs [9], vector control remains the most efficient strategy for the sustainable management of these diseases [10]. The sterile insect technique (SIT) is one control tactic that may be used as part of an area-wide integrated pest management (AW-IPM) program against tsetse fly populations [11, 12].

The SIT was successfully used as part of an AW-IPM strategy to sustainably eradicate a population of *G. austeni* from the Island of Unguja, Zanzibar in the 1990’s [13] and allowed the eradication of tsetse flies from the agro-pastoral land in Sidéradougou, Burkina Faso and in Jos, Nigeria [14, 15]. The latter two programmes were however not sustainable, as they were not implemented following AW-IPM principles, and hence suffered from re-invasion of wild flies from neighbouring areas.

The integration of the SIT in AW-IPM strategies to manage populations of tsetse flies requires the production of large numbers of high quality sterile males that are released in the target area to compete with wild males for matings with wild females of the targeted species. The mass production of the required males will depend on the successful establishment and maintenance of a large, healthy colony of the targeted species in large production facilities. In some tsetse species such as *Glossina pallidipes*, colonies that are infected with a hytrosavirus, the salivary gland hypertrophy virus (SGHV), suffer from low male and female fertility which makes the maintenance of these colonies very difficult or even impossible [16–18]. This obviously hampers the implementation of AW-IPM programmes that have an SIT component. Tsetse colonies of species that are susceptible to the negative effects of the SGHV require the implementation of some measurements to manage the virus infection to enable colony maintenance and growth [19, 20].

The successful establishment of a large colony of *G. pallidipes* will not only depend on the virus infection but can also be affected by the tsetse associated symbiotic bacteria. Tsetse flies harbour four main symbiotic bacteria: (i) *Wigglesworthia glossinidia*, an obligate symbiotic bacterium that is present in all tsetse species. Its removal from a tsetse fly using antibiotic supplements in the tsetse’s diet results in the loss of fertility [21–23], (ii) The commensal *Sodalis glossinidius*, present in all individuals of laboratory-maintained tsetse lines but not abundant in natural populations. It has been detected in the haemolymph, salivary glands and milk gland of the tsetse fly but also in the midgut where it lives in close proximity with trypanosomes [24–26], (iii) *Wolbachia*, which is an obligate intracellular and maternally transmitted alpha-proteobacterium that infects many arthropod and filarial nematode species [27, 28]. *Wolbachia* is responsible for the induction of a number of reproductive alterations and cytoplasmic incompatibility (CI) [27, 28]. *Wolbachia* infections occur in some tsetse fly species, both in the laboratory and in nature. Available data indicate that *Wolbachia* infections were heterogeneous in the field, ranging from 0 to 100% in natural populations of *G. austeni* and *G. brevipalpis* and from 9.5 to 100% in natural populations of *G. m. morsitans* [29]. It has been reported that the presence of *Wolbachia* is associated with reduced prevalence of infections with pathogenic viruses and *Plasmodium* [30–40]. Therefore, the presence of *Wolbachia* in tsetse species might also reduce trypanosome and SGHV infections and transmission, and (iv) *Spiroplasma* that was recently detected in *G. fuscipes* and *G. tachnoides* but its impact on tsetse fly performance remains unclear [41].

In support of the potential development of sustainable AW-IPM strategies that might include an SIT component against tsetse species in West Africa, we assessed the prevalence of trypanosomes, SGHV and *Wolbachia* in a large number of wild specimens from five countries as well as the potential interactions among these three microbes.

## Methods

### Sampling tsetse

Adult tsetse flies of *G. palpalis gambiensis, G. tachinoides, G. morsitans submorsitans,* and *G. medicorum* were collected between 2008 and 2015 in 46 geographical locations from five countries in West Africa (Burkina Faso, Guinea, Ghana, Mali, Senegal) (Tables 1 and 2). The flies were collected using the biconical Challier-Laveissière trap [42] and the monoconical Vavoua trap [43, 44] set as previously described [45]. On average, 20 traps were deployed per location to collect a minimum of 10 adult flies per location that were sorted by species and sex [46]. Collected flies were preserved in 95% ethanol, labeled and shipped to the FAO/IAEA Insect Pest Control Laboratory (IPCL) in Seibersdorf, Austria where they were stored at − 20 °C until further use. Species status was confirmed using molecular identification tools including internal transcribed spacers (ITS), mitochondrial DNA cytochrome oxidase subunit 1 and microsatellites (Augustinos 2018 this special issue).

**Table 1:** List of collections of tsetse adults that were analyzed to establish the prevalence of Trypanosomes, *Wolbachia* and Salivary gland hypertrophy virus (SGHV) in wild tsetse populations in West African countries

Country	No. of locations	No. of collected flies	Collection year
Burkina Faso	10	2062	2008, 2010, 2013, 2015
Mali	10	364	2008, 2010, 2011, 2012, 2013
Senegal	7	128	2008
Ghana	11	234	2008
Guinea	8	314	2008, 2009
Total	46	3102

**Table 2:** Geographic coordinates of tsetse collected samples

Glossina species	Country (area)	Longitude	Latitude
*G. tachinoides*	Burkina Faso (Folonzo)	− 4.60801757	9.92967851
	Burkina Faso (Sissili)	−2.098178	11.09447
	Burkina Faso (Comoe)	− 4.58976269	9.89106718
	Burkina Faso (Arly)	−1.289104	11.612917
	Ghana (Bougouhiba)	−0,719,172,226	10,23,885,694
	Ghana (Walewale)	−0.79846	10.351613
	Ghana (Mortani)	−0,714,119,074	10,23,479,058
	Ghana (Fumbissi)	−1,386,834,989	10,47,282,856
	Ghana (Sissili Bridge)	−1,319,208,122	10,33,035,865
	Ghana (Grogro)	−1.883133222	10.08224767
	Ghana(Kumpole)	−1,270,183,374	10,25,432,141
	Ghana (Nabogo)	−0,979,001,606	9,692,628,234
	Ghana (Psikpé)	−1,081506423	10,44,471,897
*G. palpalis gambiensis*	Burkina Faso (Kénédougou)	−4.80305222	10.98166737
	Burkina Faso (Moussodougou)	−4.95	10.833333
	Burkina Faso (Folonzo)	−4.60801757	9.92967851
	Burkina Faso (Comoé)	−4.58976269	9.89106718
	Burkina Faso (Kartasso)	−5.253033	11.141786
	Burkina Faso (Bama)	−4.4	12.033333
	Sénégal (Tambacounda)	−13.667222	13.7768889
	Sénégal (Fleuve Gambi)	−13,23,552,282	13,02433926
	Sénégal (Mako)	−13,27,338,336	12,85,430,818
	Sénégal (Niokolo)	−13,16,964,933	13,06555831
	Sénégal (Fleuève Gambi)	−12,35,811,122	12,84,670,702
	Sénégal (Diaguiri)	−12,09137828	12,62,932,251
	Sénégal (Moussalla)	−17,37,981,432	12,9,297,035
	Mali (Baoule)	−8.62	12.88
	Mali (Banko)	−6.516667	12.1
	Mali (Siby)	−8.32664	12.377685
	Mali (Système Sénégal)	−11.103663	13.416551
	Mali (Système Niger)	−4.201945	14.466284
	Mali (Bani)	−4,202,017	14,466,353
	Mali (Bougouni)	−7.483333	11.416667
	Mali (Sikasso)	−5.666667	11.316667
	Mali (Kita)	−9.484723	13.04114
	Mali (Baguineda)	−7.776667	12.615278”
	Guinea (Kangoliya)	−13.65584	9.96084
	Guinea (Dekonkore)	−10.016667	9.85
	Guinea (Bafing)	−7.524724	8.325205
	Guinea (Lemonako)	−11.566667	11.733333
	Guinea (Kerfala)	−9.461194	11.343966
	Guinea (Mimi)	−9.053083	10.400434
*G. morsitans submorsitans*	Burkina Faso (Folonzo)	−4.60801757	9.92967851
	Burkina Faso (Sissili)	−2.098178	11.09447
	Burkina Faso (Comoe)	−4.58976269	9.89106718
*G. medicorum*	Burkina Faso (Comoe)	−4.58976269	9.89106718
	Burkina Faso (Folonzo)	−4.60801757	9.92967851

### DNA extraction

The flies were removed from ethanol and rehydrated in distilled water. The wings and legs were removed for other studies. The total DNA was extracted from the remaining fly body using the DNeasy tissue kit (QIAGEN Inc., Valencia, CA) following the supplier’s instructions and was eluted in 200 μl elution buffer. All the extracted DNA samples from these locations were tested for a tsetse-specific sequence to confirm the quality.

### PCR amplification and prevalence analysis

#### SGHV prevalence

Polymerase chain reactions (PCR) were used to amplify the partial coding regions of two conserved putative ORFs, odv-e66 and dnapol (GenBank accession numbers: EF568108) using *Glossina pallidipes* Salivary Hypertrophy Virus (GpSGHV)-specific primers [47]. These primers were used in a multiplex PCR, and all the samples included a set of specific primers amplifying the *G. pallidipes* microsatellite GpCAG133 sequence to control the quality of the extracted DNA [48]. For all PCR amplifications, 22.5 μl of 1.1× Pre-Aliquoted PCR Master Mix (ABgene, UK) was used. A final volume of 25 μl of this mix contained: 0.625 units Thermoprime Plus DNA Polymerase, 75 mM Tris–HCl (pH 8.8 at 25 °C), 20 mM (NH_4_)_2_SO_4_, 2.0 mM MgCl_2_, 0.01% (*v*/v) Tween-20 and 0.2 mM each of the dNTPs. To the mix, 1.5 μl of template DNA plus forward and reverse primers were added to a final concentration of 0.2 mM per primer. Samples were considered virus-infected if any of the expected viral PCR product amplicons were detected. Data were accepted only if the control gene GpCAG133 sequence was amplified.

### Trypanosome prevalence and genotyping

For trypanosome detection, PCR was used according to Njiru et al., [8], using trypanosome specific primers to amplify the internal transcribed spacer 1 (ITS-1). PCR conditions were: 25 μl volume containing 12.5 μl of Taq PCR Master Mix kit (Qiagen) (with 0.8 Units of Taq DNA polymerase, 1.5 mM MgCl_2_, 200 μM dNTP), 0.8 μM each of the ITS-1 forward (5’-CCGGAAGTTCACCGATATTG-3′) and reverse (5’-TGCTGC GTTCTTCAACGAA- 3′) primers (VBC, Biotech, Austria), 9 μl of sterile water and 2.5 μl of genomic DNA. Cycling conditions were: 94 °C for 15 min, 94 °C for 30 s, 60 °C for 30s, 72 °C for 30s, 40 cycles following by 72 °C for 5 min; PCR products were detected by agarose (2%) gel electrophoresis and ethidium bromide staining. The sample was considered infected with trypanosome by detecting single, double or triple bands ranging from 200 bp to 700 bp (see below). DNA from *T. congolense savannah* was used as positive control which gives a PCR amplicon of 650 bp.

To have better specific and sub-specific identification of the detected trypanosomes, positive samples from the first screen were amplified with ITS-1 forward (5’-TGTAGGTGAACCTGCAGCTGGATC-3′) and ITS-1 reverse (5’-CCAAGTCATCCATCGCGACACGTT- 3′) primers following Fikru et al. [49]. The detection of different trypanosomes was based on the length of the amplicon, i.e., *T. vivax* (200 bp), *T. equiperdum*, *T. evansi* and *T. brucei* (350 bp), *T. theileri* (450 bp) and *T. congolense* savannah type (650 bp). DNA from *T. congolense* savannah type, *T. vivax*, *T. theileri*, *T. brucei gambiense*, *T. brucei rhodesiense*, *T. brucei brucei*, *T. evansi* and *T. equiperdum* provided by Dr. Stijn Deborggraeve were used as positive control.

### *Wolbachia* prevalence

PCR reaction with *Wolbachia* specific primers was used to screen the DNA of the wild tsetse flies for the presence of *Wolbachia*. The detection was based on the *Wolbachia* 16S *rRNA* gene and results in the amplification of a 438 base-pair long DNA fragment with the *Wolbachia* specific primers wspecF and wspecR [29]. The PCR conditions used were as described above for the trypanosome detection and the cycling conditions were: 94 °C for 2 min, 94 °C for 30 s, 55 °C for 30s, 72 °C for 30s, 36 cycles following by 72 °C for 5 min. As a positive control for *Wolbachia*, DNA extracted from the Mediterranean fruit fly, *Ceratitis capitata* strain S 10.3 was used. This strain is transinfected with the *w*Cer4 *Wolbachia* strain of *Rhagoletis cerasi* [50].

### Data analysis

The data were analyzed with the software package R, using a generalized linear model (GLM) with the package stat [51]. Trypanosome, virus and *Wolbachia* prevalence in tsetse were respectively considered as response variables, while tsetse species, sex, countries and their interactions were used as explicative variables. The best model was selected on the basis of the lowest corrected Akaike information criterion (AICc), and the significance of fixed effects was tested using the likelihood test ratio [52, 53]. Then, for each country, GLM were used to assess differences in trypanosome, virus and *Wolbachia* prevalence between localities and species. Trypanosome prevalence was compared between species by a pairwise comparison of proportions with a Bonferroni correction (package stats). Correlations between the prevalence of trypanosome species, salivary gland hypertrophy virus and *Wolbachia* were tested using the “rcorr” function of the Hmisc (Harrel miscellaneous package version 4.03, 2017).

## Results

### Global trypanosome prevalence

**Fig. 1:**
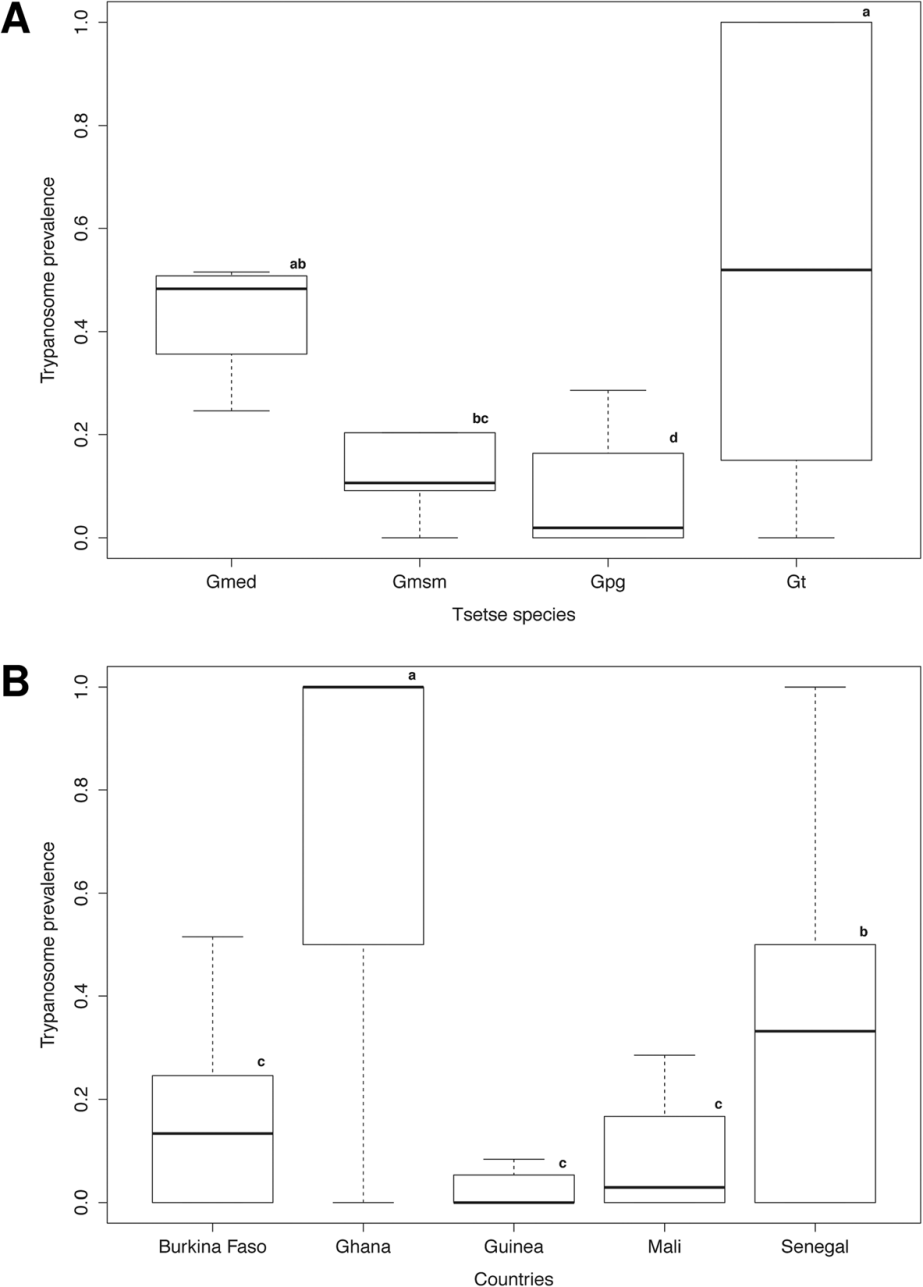
Global prevalence of trypanosomes according to tsetse species (**A**) and country (**B**). Boxes extend between the 25th and 75th percentile. A thick line denotes the median. The whiskers extend up to the most extreme values. Gmed: *Glossina medicorum*, Gmsm: *G. morsitans submositans*, Gpg: *G. palpalis gambiensis* and Gt: *G. tachinoides.* Different letters indicate significant difference. Different letters indicate significant difference

The trypanosome prevalence varied significantly from one country to another and from one species to another. Overall, 18.4% of the examined tsetse flies (*n* = 3102) were positive for trypanosomes, irrespective of tsetse species or country (Table 3). Trypanosomes were detected in *G. tachinoides* in Burkina Faso and Ghana; *G. p. gambiensis* in Burkina Faso, Guinea, Mali, and Senegal; *G. m. submorsitans* and *G. medicorum* in the Comoé forest in the south of Burkina Faso at the border with Côte d’Ivoire. The best model (lowest AICc) selected for the overall trypanosome prevalence retained the tsetse species and countries as variables that fitted well the data with no interaction. For tsetse species, *G. medicorum* (only caught in Folonzo village, and a protected area belonging to the village in Southern Burkina Faso) had the highest mean infection rate of 39.6% (Fig. 1A), which was significantly higher than the mean infection rate in *G. p. gambiensis* (*P <* 0.001). The mean trypanosome infection rate in *G. tachinoides* was also significantly higher as compared with *G. m. submorsitans* (*P* = 0.008; Fig. 1A; Additional file 1).

**Table 3:** Prevalence of trypanosomes, salivary gland hypertrophy virus and *Wolbachia* in tsetse tested samples

Species	Country	*Trypanosomes*	Virus	*Wolbachia*
*G. tachinoides*	Burkina Faso	(140/834) 16.79%	(25/834) 3%	(2/834) 0.24%
	Ghana	(143/234) 61.11%	(0/234) 0%	(0/234) 0%
*G.p.gambiensis*	Burkina Faso	(77/731) 10,53%	(14/731) 1,92%	(1/731) 0,14%
	Mali	(25/364) 6,87%	(15/364) 4,12%	(16/364) 4,40%
	Guinea	(7/314) 2,23%	(0/314) 0%	(13/314) 4,14%
	Senegal	(58/128) 43,75%	(0/128) 0%	(0/128) 0%
*G. m. submorsitans*	Burkina Faso	(62/343) 18.08%	(4/343) 1,17%	(1/343) 0,29%
*G. medicorum*	Burkina Faso	(61/154) 39.61%	(1/154) 0,65%	(1/154) 0,65%
Total		(570/3102) 18,38%	(54/3102) 1,74%	(30/3102) 0,96%

Trypanosome prevalence by country was low in Guinea (2.2%) and Mali (6.9%) but high in Senegal (43.7%) and Ghana (61.1%) (Table 3). The result showed no significant difference between the trypanosome prevalence in Burkina Faso, Guinea and Mali but the prevalence of these three countries was significantly different from that of Senegal and Ghana (*P* < 0.05) (Fig. 1B and Additional file 1). The sex effect was not retained in the model highlighting no difference in the mean prevalence of male and female flies. All *G. tachinoides* flies collected from Fumbissi (*n* = 15), Grogro (*n* = 11), Kumpole (*n* = 7), Psikpé (*n* = 2) and Sissili Bridge (*n* = 6) in Ghana were infected with trypanosomes, and the overall prevalence in seven out of nine locations was relatively > 53% (Table 4). Trypanosome prevalence in the other tsetse species fluctuated greatly with location, i.e., from 0% in the *G. p. gambiensis* flies collected in Comoé, Kenedougou and Bama to 34.5% in Moussodougou in Burkina Faso (Table 5). A similar trend was found in *G. p. gambiensis* flies collected in Mali and Guinea.

**Table 4:** Trypanosome prevalence in natural populations of *Glossina tachinoides* collected from Ghana

Location	Sample size	Prevalence
Bougouhiya	19	(3/19) 15.78%
Fumbissi	15	(15/15) 100%
Grogro	11	(11/11) 100%
Kumpole	7	(7/7) 100%
Mortani	41	(22/41) 53.65%
Nabogo	2	(0/2) 0%
Psikpé	2	(2/2) 100%
Sissili Bridge	6	(6/6) 100%
Walewale	131	(77/131) 58.77%
Total	234	(143/234) 61.11%

**Table 5:** Trypanosome prevalence in natural populations of *Glossina palpalis gambiensis* collected from Burkina Faso

Location	Sample size	Prevalence
Bama	77	(0/77) 0%
Comoé	123	(3/123) 2.43%
Folonzo	237	(27/237) 11.39%
Kartasso	136	(0/136) 0%
Kenedougou	41	(0/41) 0%
Moussodougou	142	(49/142) 34.50%
Total	731	(77/731) 10.53%

### Prevalence of different trypanosome species in wild populations of tsetse in Western Africa

**Fig. 2:**
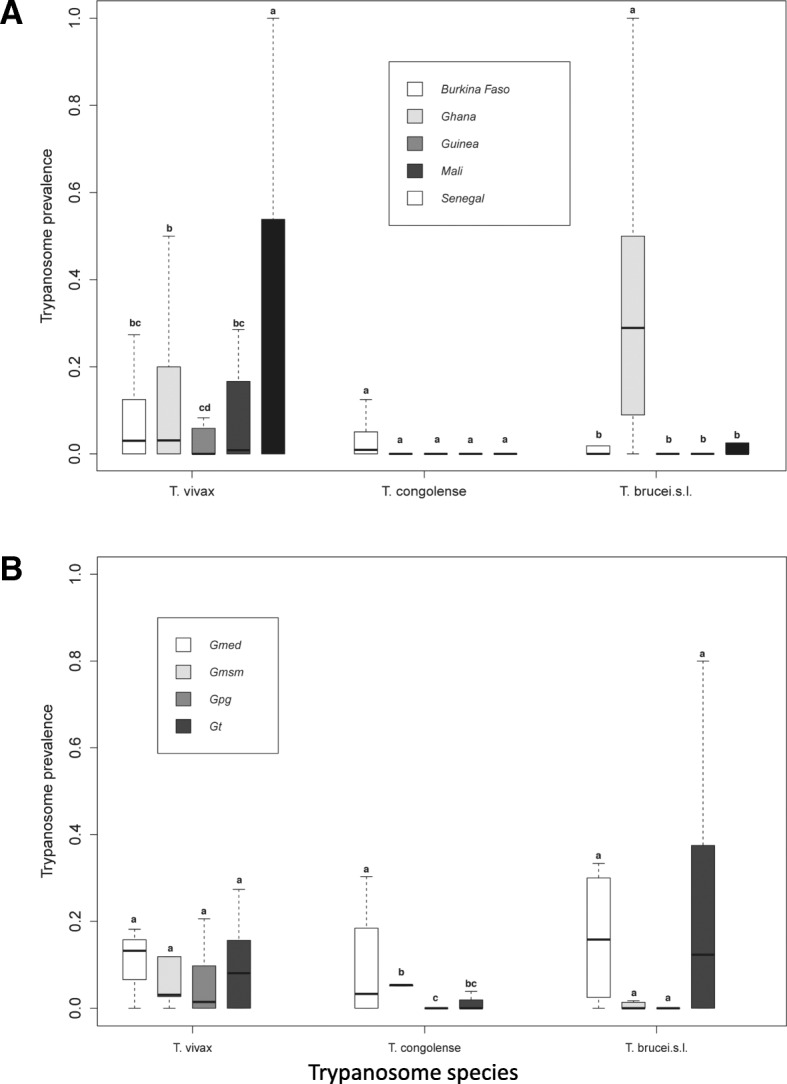
**a** Prevalence of *Trypanosoma vivax, Trypanosoma congolense* and *Trypanosoma spp* single infection according to country (**a**) and tsetse species (**b**). Boxes extend between the 25th and 75th percentile. A thick line denotes the median. The whiskers extend up to the most extreme values. Gmed: *Glossina medicorum*, Gmsm: *G. morsitans submositans*, Gpg: *G. palpalis gambiensis* and Gt: *G. tachinoides.* Different letters indicate significant difference of trypanosome infection prevalence between counties (**A**) and tsetse species (**B**)

The results indicate that tsetse flies in West Africa could be infected with different species of trypanosomes in single or multiple infections. For *T. vivax* prevalence the best model retained countries as variable that fitted well the data indicating that the prevalence of *T. vivax* alone, did not differ significantly among tsetse fly species and sex (Additional file 1) but the mean infection rate of *T. vivax* in Senegal was significantly higher as compared to other countries (*P* < 0.05), also the prevalence in Ghana was significantly higher as compared to Guinea (*P* = 0.030; Fig. 2; Additional file 1).

GLM results for single infections with *T. congolense* selected for species as variable that fitted well the data indicated that the prevalence of *T. congolense* alone did not differ significantly among countries and sex (Fig. 2). The *T. congolense* infection rate in *G. medicorum* was significantly higher as compared to *G. tachinoides*, *G. p. gambiensis* and *G. m. submorsitans* (*P* < 0.05); Additional file 1). *T. congolense* infection rate in *G. p. gambiensis* was significantly lower as compared to *G. m. submorsitans* (Fig. 2B; Additional file 1).

**Fig. 3:**
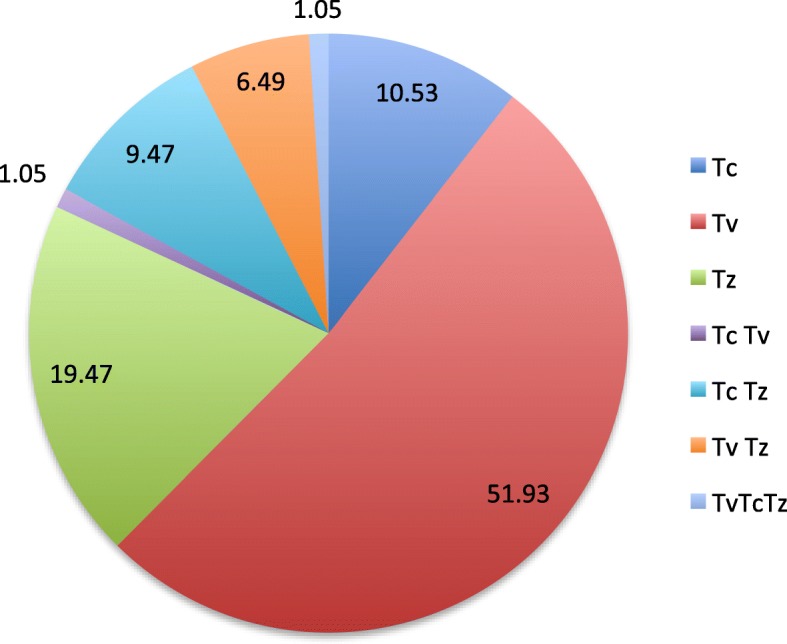
Prevalence of Trypanosome single and mixed infection if different tsetse species collected from west Africa

**Fig. 4:**
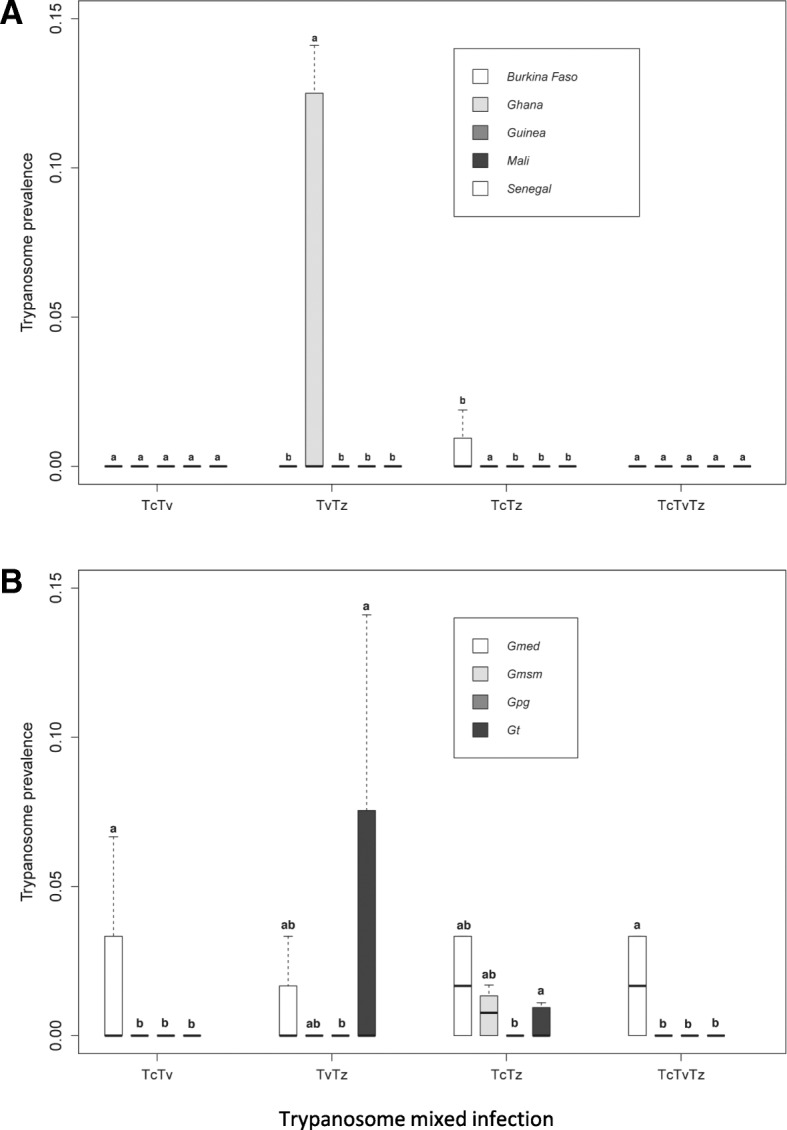
Prevalence of Trypanosome coinfection according to the country (**A**) and tsetse species (**B**). Boxes extend between the 25th and 75th percentile. A thick line denotes the median. The whiskers extend up to the most extreme values. Gmed: *Glossina medicorum*, Gmsm: *G. morsitans submositans*, Gpg: *G. palpalis gambiensis* and Gt: *G. tachinoides.* Tv*: Trypanosoma vivax*, Tc: *Trypanosoma congolensis* and Tz: *Trypanosoma spp (T. brucei*, *T. evansi*, *T. equiperdum* and *T. theileri).* Different letters indicate significant difference of trypanosome mixed infection prevalence between counties (**A**) and tsetse species (**B**)

Non-specific detection of *Trypanosoma* spp*.* (*Tz*) (including *T. brucei*, *T. evansi*, *T. equiperdum* and *T. theileri)* based on the primer detection was recorded in 19.4% of the samples (Fig. 3). Results model selected for countries as variable that fitted well the data indicating that the prevalence of *Trypanosoma* spp. did not differ significantly among countries and sex. The *Trypanosoma* spp. prevalence in Ghana was significantly higher than the other countries (*P* < 0.001; Fig. 4A; Additional file 1).

Analysis of the data with the well fitted model indicated that the coinfection of *T. congolense* with *T. vivax* did not differ between countries and sex. However, *T. congolense* and *T. vivax* coinfection was significantly higher in *G. medicorum* (1.1%) as compared with the other tsetse species (*P* = 0.001; Fig. 4B; Additional file 1). The coinfection rate of *T. vivax* and other *Trypanosoma spp* in Ghana was significantly higher than all other countries (*P* < 0.01, Fig. 4A; Additional file 1). Analysis of coinfection of *T. congolense* and other *Trypanosoma spp* (7.5%) indicated that the infection rate in Ghana was significantly higher than Burkina Faso (*P* < 0.01, Fig. 4A; Additional file 1).

Analysis of triple infection of *T. vivax*, *T. congolense* with other *Trypanosoma spp* selected for species as variable that fitted well the data indicating that the prevalence of *Trypanosoma* spp. did not differ significantly among countries and sex. The infection rate in *G. medicorum* (1.1%) was significantly higher than in the other tsetse species (0%) (*P* < 0.001; Additional file 1).

### SGHV prevalence

**Fig. 5:**
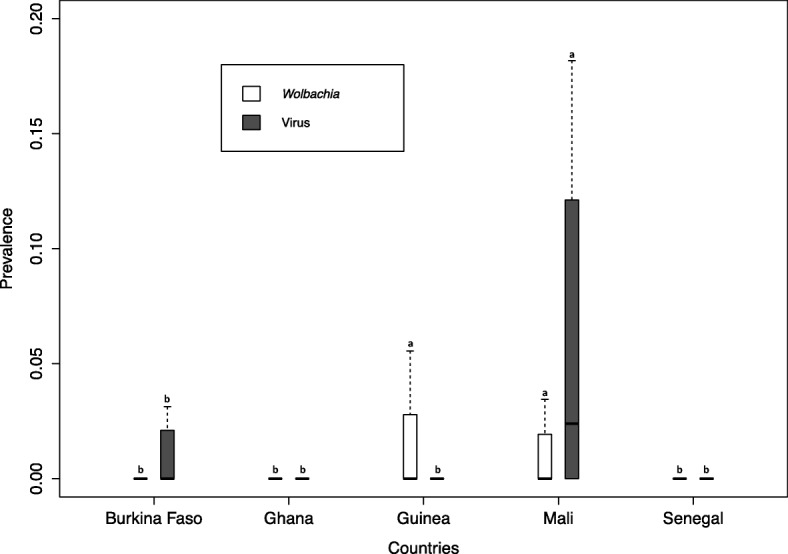
Prevalence of Salivary gland hypertrophy virus (SGHV) and *Wolbachia* according to the country (**A**) and tsetse species (**B**). Boxes extend between the 25th and 75th percentile. A thick line denotes the median. The whiskers extend up to the most extreme values. Gmed: *Glossina medicorum*, Gmsm: *G. morsitans submositans*, Gpg: *G. palpalis gambiensis* and Gt: *G. tachinoides.* Different letters indicate significant difference

Based on the PCR screen used in the present study, the average prevalence of SGHV in all collected flies was 1.7% (*n* = 54) (Table 2). The prevalence varied from 0% in *G. tachinoides* samples from Ghana and *G. p. gambiensis* samples from Senegal and Guinea to 4.1% in *G. p. gambiensis* flies from Mali (Fig. 5). The result indicated that the SGHV prevalence did not differ significantly among species and sex. However, the virus prevalence was significantly higher in Mali compared with the other countries (*P* = 0.001; Additional file 1).

### Prevalence of *Wolbachia*

The prevalence of *Wolbachia* was low in all tested species and averaged at 1.0% (Table 3). The prevalence did not differ significantly among species and sex. The *Wolbachia* prevalence in Mali was significantly higher as compared to Senegal, Ghana and Burkina Faso (*P* < 0.05; Additional file 1). No other significant difference was observed (Fig. 4).

### Mixed infection of trypanosomes, SGHV and *Wolbachia*

The prevalence data indicate that the mean trypanosome infection rate was higher as compared with the prevalence of the SGHV and *Wolbachia*. Most of the flies (99.94%) that were infected with trypanosomes were negative for *Wolbachia*. In *G. tachinoides* and *G. m. submorsitans*, double infection with SGHV and trypanosomes was observed at a low prevalence, i. e. 0.5% and 0.4% respectively. No double infection of SGHV and trypanosome was detected in *G. p. gambiensis*. The *Trypanosoma* spp. infection rate was significantly positively correlated with that of the virus (*P* < 0.001), although the correlation was weak (*r* = 0.45). No significant correlation was observed between *Wolbachia* and SGHV.

### Impact of tsetse fly gender on trypanosomes, SGHV and *Wolbachia* prevalence

There was no significant difference between male and female infection by trypanosomes (*P* = 0.377), SGHV (*P* = 0.739) or *Wolbachia* (*P* = 0.362).

### Trypanosomes, SGHV and *Wolbachia* distribution per countries

Burkina Faso showed the highest species diversity with four tsetse species collected: *G. p. gambiensis*, *G. tachinoïdes*, *G. m. submorsitans* and *G. medicorum*. Among the ten localities sampled, these four species were found together in Folonzo and Comoe. *G. p. gambiensis* flies were found in four other localities: Bama, Kartasso, Kenedougou and Mousodougou. *G. tachinoïdes* and *G. m. submorsitans* flies were found together in Sissili, however, in Arly *G. tachinoïdes* only was found (Table 2). Flies infected with trypanosomes were found in five localities. *Trypanosoma vivax* prevalence was not different between localities and species (Additional file 1). For *T. congolense* no differences between localities were highlighted. However, significant differences were observed between tsetse species. *G. medicorum* was the most infected species (9%) and was different from all other species (*G. m. submorsitans* 5.2%; *G. tachinoïdes* 2.4% and *G. p. gambiensis* 0.4%; Additional file 1). For *Trypanosoma* spp., significant differences were observed between tsetse species in Comoe and Folonzo. In both localities, *G. medicorum* (3.2% and 30% respectively) was significantly more infected than *G. m. submorsitans* (0.4% and 0.7% respectively) and *G. tachinoïdes* (0.2% and 1.5%) (Additional file 1). Flies infected with SGHV were found in four localities. No difference between tsetse species and localities was observed (Additional file 1). *Wolbachia* prevalence was not different between species. Tsetse flies (*G. tachinoïdes*, *G. p. gambiensis*, *G. medicorum* and *G. m. submorsitans*) from two localities were infected with *Wolbachia*. *Wolbachia* prevalence in tsetse flies from Kenedougou was significantly more important than Comoe (2.4% and 0.5% respectively).

In Mali, flies from only one tsetse species (*G. p. gambiensis)* were collected in the ten localities sampled. *T. vivax* infection was found in seven localities and the prevalence in Baoule (42.8%) was significantly higher than the others (Bagnuineda 16.6%, Banko 21.9%, Bani 1.4%, Kita 16.6%, Système Niger 1.1%, Système Sénégal 2%; Additional file 1). *T. congolense* was only found in Système Niger (1.1%) and *Trypanosoma* spp. in Sikasso (3.4%) and Système Niger (2.3%) with no differences. SGHV was found in the ten localities of Mali and *Wolbachia* in four without any differences (Additional file 1).

In Senegal, only *G. p. gambiensis* were found between the seven localities sampled. *T. congolense* infection was not found, however *T. vivax* infection was found in five localities (Mako, Fleuve G, Fleuve Gambie, Niokolo and Tambacounda) and *Trypanosoma sp* in two (Diaguiri and Tambacounda). No significant differences in trypanosome prevalence were found between different localities (Additional file 1). No SGHV and *Wolbachia* were found in tsetse flies analysed.

In Ghana, *G. tachinoides* was the only species caught among the eleven localities sampled and eight of them were found positive for trypanosomes. For *T. vivax,* significant differences in trypanosome prevalence were found between localities. The locality of Grogro showed the highest prevalence (36%) and was significantly different from all localities except Fumbissi. On the contrary, the locality of Bougouhiya showed the lowest prevalence (0.05%) and was significantly different from Fumbissi, Grogro and Kumpole. Fumbissi was also different from Mortani, Sissili bridge and Walewale (Additional file 1). *T. congolense* was only found in one locality: Walewale. *Trypanosoma* spp. was found in the eight positive localities. Among these, flies collected at the localities of Kandiaga and Sissili bridge were the most infected (100% and 83% respectively) and were significantly different from all others but not between them. No virus and *Wolbachia* were found.

In Guinea, *G. tachinoides* was the only species caught from all localities. Out of eight localities sampled, tsetse flies collected from six of them were found positive for trypanosomes. *T. congolense* and *Trypanosoma* spp. were not found and no significant difference in trypanosome prevalence for *T. vivax* was observed (Additional file 1). SGHV was absent and *Wolbachia* was found in three localities but no difference in prevalence was observed (Additional file 1).

## Discussion

The results of this study indicate an overall low prevalence of SGHV and *Wolbachia* and a high prevalence of trypanosomes in the sampled wild tsetse populations. The prevalence of all three microbes varied between species and between locations but there was no significant difference between male and female flies. All flies sampled in Kimpole (100%), Grogro (100%), Fumbissi (100%), Sissili Bridge (100%) and Psikpe (100%) of Ghana were infected with trypanosomes, an infection rate that was significantly higher as compared to other locations. In some cases, mixed infections with different trypanosome species were detected, as well as mixed infections of trypanosomes and SGHV. However, no mixed infection of trypanosomes or SGHV with *Wolbachia* was detected.

The method of detection and characterization of the type of trypanosome infection using the ribosomal internal transcribed spacer (ITS) is known to be sensitive and it provides quick information about the trypanosome type circulating in the infected area. However, these identified trypanosomes may not be the only ones circulating within the different areas as was observed in Guinea. Other types of trypanosome species may also be circulating but due to the lack of PCR primers cannot be identified [54]. In addition, Pagabeleguem et al. [55] noted that the trypanosome infection rate in tsetse flies was always higher by microscopy than PCR and suggested that almost half of the flies were infected by trypanosome species non-pathogenic for cattle.

The relatively high frequency of pathogenic trypanosomes in tsetse was previously linked to high AAT prevalence in cattle, especially in the locality of Folonzo in Burkina Faso [55]. It has therefore been suggested that the detection of trypanosome infection in tsetse flies might provide indirect information about the AAT prevalence in livestock in the selected area and hence the potential risk of uninfected animals to become infected. This may not be so relevant for HAT as the link between tsetse infection and disease prevalence is considered to be weak. In Guinea, for example, *T. brucei gambiense* is the pathogenic trypanosome identified in humans, while no *T. brucei gambiense* infection has been found in tsetse confirming the usual very low (0.1%) mature infection rates of *T. brucei gambiense* in tsetse, even in active sleeping sickness foci [56].

The SGHV was reported in *G. p. palpalis* in Côte d’Ivoire in 1978 at a very low prevalence (0.3%) [57]. Although the prevalence of SGHV based on fly dissection was generaly low in wild tsetse populations (0.5–5%) [58], the prevalence detected by PCR can be very high (100%) [47]. These results clearly indicate that the SGHV prevalence in tsetse species in West Africa is significantly lower than the SGHV prevalence in *G. pallidipes* in eastern and southern Africa previously reported [47], where the virus prevalence varied from 2 to 100%, depending on the location. However, the low virus prevalence in West African tsetse populations might be underestimated due to the primer specificity and the sensitivity of the PCR, as all primers were based on the nucleotide sequence of *G. pallidipes* SGHV. A different virus sequence in other tsetse species in West Africa would then result in a lower detection rate. To overcome this problem, it is suggested to have the entire genome sequenced of each virus detected in each tsetse species to enable the selection of more specific and sensitive primers for virus detection.

*Wolbachia* is known to be present in wild tsetse populations [29, 59], and using standard PCR assays, it was detected in *G. m. morsitans*, *G. m. centralis* and *G. austeni* populations, but not in *G. tachinoides*. Using alternative assays *Wolbachia* was also detected at low infection rates in *G. fuscipes* and *G. morsitans* subspecies [59, 60]. The prevalence of *Wolbachia* in *G. p. gambiensis* from Burkina Faso was very low (~ 0.14%) In *G. m. morsitans* the prevalence of *Wolbachia* was higher and varied between 10 and 100% depending on the location [51]. In *G. f. fuscipes* collected from Uganda, the prevalence of *Wolbachia* varied between 26 and 55%, which is higher than the prevalence reported in this study [29]. It is important to note that in the study of Alam and colleagues the detection method used for screening the *Wolbachia* infection was the sequential PCR method (high sensitivity but low specificity). In this study and in the study of Doudoumis and colleagues, a traditional one step PCR was used for the detection [29, 59] to avoid any non-specific detection and to detect only high level *Wolbachia* infections that might interfere with the virus and trypanosome infection. We also tried to avoid detecting *Wolbachia* chromosomal insertions by using primers specific for active *Wolbachia* in the cytoplasm [29, 61]. Presence of extensive *Wolbachia* insertions was discovered in the genome of its host *G. m morsitans* [61]. The low prevalence of *Wolbachia* detected in wild tsetse populations in this study might be due to (i) the absence of *Wolbachia* infection, (ii) the low titer of *Wolbachia* infection or (iii) the presence of another *Wolbachia* strain that cannot be detected with the primers used in this study.

Mixed infections of trypanosomes, SGHV and *Wolbachia* have been previously reported [59] and this was also the case in our study, although the correlation was low (*r* = 0.45; *P* < 0.001). In the study of Alam et al. [59], the author mentioned the potential negative relationship between *Wolbachia* and SGHV infection, which was also observed in our study. Trypanosome infection was found in flies that were also infected with the SGHV but no flies that were infected with *Wolbachia* showed a trypanosome infection. This suggests that the presence of *Wolbachia* might mediate the presence of different pathogens and parasites, as previously described [36, 62, 63]. Due to the low prevalence of *Wolbachia*, no possible correlation between the *Wolbachia* infection and the trypanosomes and/or SGHV could be found. On the other hand, a negative impact of trypanosome infection on *Wolbachia* presence cannot be excluded. However, these antagonistic relationships need further investigation and statistical analysis. If the assumption that *Wolbachia* might block trypanosome transmission is correct, these novel insights could be useful for the development and implementation of sterile insect technique-based population control strategies, e.g. releasing *Wolbachia*-infected males that both induce cytoplasmic incompatibility when mated with wild *Wolbachia*–free females and being refractory for trypanosome infection and transmission in a way similar to that recently developed for mosquitoes [64–67].

## Conclusion

The results of this study indicate a high rate of trypanosome infection in tsetse wild populations but lower infection rate of both *Wolbachia* and SGHV. Mixed infections with different trypanosome species or trypanosome with SGHV were found. The high rate of trypanosome infection in tsetse populations might be used as an indicator of the presence of trypanosomiosis in both human and animal by determining the different trypanosomes circulation in the targeted area. The low prevalence of *Wolbachia* in tsetse flies in West Africa and the lack of mixed infection of *Trypanosoma* spp., and *Wolbachia*, which might indicate an antagonistic relationship, require further investigation. The low prevalence of SGHV in the field population is encouraging for SIT programmes as it might exclude the SGHV outbreaks in tsetse mass-rearing established from such low infected populations; however, it encourages the implementation of the virus management strategies to control the virus infection to avoid such problem.

## Additional file

Additional file 1: Generalized linear model (GLM) fixed effect statistical results. (DOC 269 kb)

## Abbreviations

AAT: Animal African trypanosomosis
AICc: Akaike information criterion
AW-IPC: Area-wide integrated pest management programs
GpSGHV: *Glossina pallidipes* salivary gland hypertrophy virus
HAT: Human African trypanosomosis
IPCL: Insect Pest Control Laboratory
ITS: Internal transcribed spacers
PBS: Phosphate Buffer Saline
qPCR: Quantitative polymerase chain reaction
SGHV: Salivary gland hypertrophy virus
SIT: Sterile Insect Technique
T: *Trypanosoma*
