# Supplementary material for: Prevalence of trypanosomes, salivary gland hypertrophy virus and Wolbachia in wild populations of tsetse flies from West Africa
Source: BMC Microbiol. 2018 Nov 23;18(Suppl 1):153. doi: 10.1186/s12866-018-1287-4 (PMC6251090; doi:10.1186/s12866-018-1287-4)
Supplement: Supplementary file 1 — Generalized linear model (GLM) fixed effect statistical results. (DOC 269 kb) [file 12866_2018_1287_MOESM1_ESM.doc]

**Additional file 1.** Generalized linear model (GLM) fixed effect statistical results.

| **Test** | **Fixed effects** | **estimate** | **SE** | **T-value** | **P-value** |
| --- | --- | --- | --- | --- | --- |
| overall trypanosome prevalence | Intercept | 0.37689 | 0.14872 | 2.534 | 0.01317 |
|  | Gpg | -0.35270 | 0.13576 | -2.598 | 0.01112 |
|  | Gt | -0.27785 | 0.14400 | -1.930 | 0.05712 |
|  | Gmsm | -0.27160 | 0.15774 | -1.722 | 0.08888 |
|  | MLI | 0.08008 | 0.08221 | 0.974 | 0.33285 |
|  | SN | 0.31893 | 0.09600 | 3.322 | 0.00134 |
|  | GH | 0.59835 | 0.13520 | 4.426 | 2.93e-05 |
|  | BKF | 0.05504 | 0.09107 | 0.604 | 0.54728 |
| overall trypanosome prevalence | Intercept | 0.424225 | 0.145590 | 2.914 | 0.00460 |
|  | Gmed | 0.271598 | 0.157743 | 1.722 | 0.08888 |
|  | Gpg | -0.081106 | 0.125168 | -0.648 | 0.51881 |
|  | Gt | -0.006252 | 0.134056 | -0.047 | 0.96291 |
|  | GN | -0.318933 | 0.095999 | -3.322 | 0.00134 |
|  | MLI | -0.238850 | 0.092744 | -2.575 | 0.01181 |
|  | GH | 0.279417 | 0.141850 | 1.970 | 0.05224 |
|  | BKF | -0.263893 | 0.100685 | -2.621 | 0.01045 |
| *Trypanosoma vivax* prevalence | Intercept | 0.09770 | 0.04212 | 2.320 | 0.02277 |
|  | GH | 0.06941 | 0.05957 | 1.165 | 0.24716 |
|  | SN | 0.22951 | 0.07048 | 3.256 | 0.00162 |
|  | BKF | -0.02289 | 0.05362 | -0.427 | 0.67055 |
|  | GN | -0.06796 | 0.06247 | -1.088 | 0.27978 |
| *Trypanosoma vivax* prevalence | Intercept | 0.32720 | 0.05651 | 5.790 | 1.15e-07 |
|  | MLI | -0.22951 | 0.07048 | -3.256 | 0.001622 |
|  | BKF | -0.25240 | 0.06553 | -3.851 | 0.000227 |
|  | GH | -0.16009 | 0.07048 | -2.271 | 0.025643 |
|  | GN | -0.29746 | 0.07295 | -4.077 | 0.000102 |
| *Trypanosoma vivax* prevalence | Intercept | 0.02974 | 0.04614 | 0.645 | 0.520920 |
|  | SN | 0.29746 | 0.07295 | 4.077 | 0.000102 |
|  | MLI | 0.06796 | 0.06247 | 1.088 | 0.279784 |
|  | GH | 0.13737 | 0.06247 | 2.199 | 0.030609 |
|  | BKF | 0.04507 | 0.05683 | 0.793 | 0.430021 |
| *Trypanosoma congolensis* prevalence | Intercept | 0.012935 | 0.006171 | 2.096 | 0.0390 |
|  | Gmed | 0.079216 | 0.016899 | 4.687 | 1.03e-05 |
|  | Gpg | -0.011808 | 0.007489 | -1.577 | 0.1185 |
|  | Gmsm | 0.029255 | 0.015365 | 1.904 | 0.0603 |
| *Trypanosoma congolensis* prevalence | Intercept | 0.04219 | 0.01407 | 2.998 | 0.00355 |
|  | Gt | -0.02925 | 0.01537 | -1.904 | 0.06026 |
|  | Gmed | 0.04996 | 0.02111 | 2.367 | 0.02018 |
|  | Gpg | -0.04106 | 0.01470 | -2.794 | 0.00642 |
| *Trypanosoma Spp (Tz)* prevalence | Intercept | 0.34998 | 0.03820 | 9.162 | 2.54e-14 |
|  | BKF | -0.31235 | 0.04863 | -6.423 | 7.36e-09 |
|  | GN | -0.34998 | 0.05666 | -6.177 | 2.17e-08 |
|  | SN | -0.29010 | 0.06392 | -4.538 | 1.85e-05 |
|  | MLI | -0.34447 | 0.05402 | -6.377 | 9.03e-09 |
| Tc-Tv prevalence | Intercept | 0.016667 | 0.003287 | 5.070 | 2.25e-06 |
|  | Gt | -0.015658 | 0.003531 | -4.435 | 2.71e-05 |
|  | Gmsm | -0.016667 | 0.004410 | -3.779 | 0.000289 |
|  | Gpg | -0.016667 | 0.003405 | -4.895 | 4.54e-06 |
| Tv-Tz prevalence | Intercept | 0.13394 | 0.02686 | 4.987 | 3.21e-06 |
|  | BKF | -0.12682 | 0.03419 | -3.709 | 0.000370 |
|  | GN | -0.13394 | 0.03984 | -3.362 | 0.001161 |
|  | SN | -0.11124 | 0.04494 | -2.475 | 0.015308 |
|  | MLI | -0.13394 | 0.03799 | -3.526 | 0.000682 |
| Tc-Tz prevalence | Intercept | 0.037894 | 0.008111 | 4.672 | 1.11e-05 |
|  | BKF | -0.032569 | 0.010325 | -3.154 | 0.00222 |
|  | GN | -0.037894 | 0.012030 | -3.150 | 0.00225 |
|  | SN | -0.037894 | 0.013572 | -2.792 | 0.00647 |
|  | MLI | -0.037894 | 0.011470 | -3.304 | 0.00140 |
| Tc-Tv-Tz prevalence | Intercept | 0.016667 | 0.003533 | 4.717 | 9.17e-06 |
|  | Gt | -0.013997 | 0.003795 | -3.688 | 0.000395 |
|  | Gmsm | -0.016667 | 0.004740 | -3.516 | 0.000702 |
|  | Gpg | -0.016667 | 0.003659 | -4.555 | 1.72e-05 |
| SGHV virus prevalence | Intercept | 0.034894 | 0.009162 | 3.808 | 0.000263 |
|  | GH | -0.034894 | 0.012957 | -2.693 | 0.008530 |
|  | SN | -0.034894 | 0.015331 | -2.276 | 0.025362 |
|  | BKF | -0.032345 | 0.011664 | -2.773 | 0.006824 |
|  | GN | -0.011395 | 0.013590 | -0.839 | 0.404099 |
| *Wolbachia* prevalence | Intercept | 0.034894 | 0.009162 | 3.808 | 0.000263 |
|  | GH | -0.034894 | 0.012957 | -2.693 | 0.008530 |
|  | SN | -0.034894 | 0.015331 | -2.276 | 0.025362 |
|  | BKF | -0.032345 | 0.011664 | -2.773 | 0.006824 |
|  | GN | -0.011395 | 0.013590 | -0.839 | 0.404099 |
| the *T.v.* prevalence in Burkina Faso | Intercept | 0.15648 | 0.05654 | 2.768 | 0.0244 |
|  | Folonzo | -0.02315 | 0.09793 | -0.236 | 0.8191 |
|  | Moussodougou | 0.11973 | 0.09793 | 1.223 | 0.2563 |
|  | Sissili | -0.02716 | 0.07996 | -0.340 | 0.7428 |
|  | Gmsm | -0.12788 | 0.07996 | -1.599 | 0.1484 |
|  | Gpg | -0.14240 | 0.09793 | -1.454 | 0.1840 |
|  | Gt | -0.03349 | 0.07996 | -0.419 | 0.6864 |
|  | Folonzo:Gmsm | 0.22720 | 0.12642 | 1.797 | 0.1100 |
|  | Folonzo:Gpg | 0.05152 | 0.13849 | 0.372 | 0.7196 |
|  | Folonzo:Gt | 0.09832 | 0.12642 | 0.778 | 0.4591 |
| *T.c.* prevalence in Burkina Faso | Intercept | 0.18430 | 0.04133 | 4.459 | 0.000964 |
|  | Gmsm | -0.13156 | 0.05062 | -2.599 | 0.024743 |
|  | Gpg | -0.17005 | 0.05336 | -3.187 | 0.008656 |
|  | Gt | -0.13781 | 0.04773 | -2.887 | 0.014777 |
| *T.z.* prevalence in Burkina Faso | Intercept | 0.030303 | 0.016419 | 1.846 | 0.124 |
|  | Folonzo | -0.025160 | 0.016419 | - 1.532 | 0.186 |
|  | Kartasso | 0.005298 | 0.016419 | 0.323 | 0.760 |
|  | Moussodougou | 0.005378 | 0.016419 | 0.328 | 0.757 |
|  | Gmsm | -0.015036 | 0.023220 | - 0.648 | 0.546 |
|  | Gpg | - 0.013645 | 0.020109 | - 0.679 | 0.528 |
|  | Gt | 0.014817 | 0.020109 | 0.737 | 0.494 |
|  | Folonzo:Gmsm | 0.036560 | 0.028439 | 1.286 | 0.255 |
|  | Folonzo:Gpg | 0.065106 | 0.025961 | 2.508 | 0.054 |
| *Wolbachia* prevalence in Burkina Faso | Intercept | 0.008062 | 0.002904 | 2.776 | 0.0692 |
|  | Kenedougou | 0.033604 | 0.006493 | 5.175 | 0.0140 |
| *T.v.* prevalence in Mali | Intercept | 0.42857 | 0.02822 | 15.184 | 0.00431 |
|  | Baguinega | -0.26190 | 0.04889 | - 5.357 | 0.03312 |
|  | Banco | - 0.18277 | 0.03992 | - 4.579 | 0.04453 |
|  | Bani | -0.41133 | 0.04889 | - 8.414 | 0.01383 |
|  | Kita | - 0.26190 | 0.04889 | - 5.357 | 0.03312 |
|  | SN | - 0.40934 | 0.04889 | - 8.373 | 0.01397 |
|  | SS | - 0.38857 | 0.04889 | - 7.948 | 0.01546 |
| *SGHV.* prevalence in Mali | Intercept | 0.16667 | 0.05889 | 2.830 | 0.0473 |
|  | Banco | -0.13725 | 0.08328 | - 1.648 | 0.1747 |
|  | Bani | 0.0671 | 0.07212 | - 0.931 | 0.4046 |
|  | Bougouni | -0.01190 | 0.07212 | - 0.165 | 0.8769 |
|  | Sikasso | -0.04545 | 0.08328 | - 0.546 | 0.6142 |
|  | SN | -0.14277 | 0.07212 | - 1.979 | 0.1189 |
|  | SS | -0.12583 | 0.07212 | - 1.745 | 0.1560 |
|  | Intercept | 0.16667 | 0.05889 | 2.830 | 0.0473 |
| *Wolbachia* prevalence in Mali | Intercept | 0.14286 | 0.09085 | 1.572 | 0.361 |
|  | Bani | - 0.10837 | 0.12848 | - 0.843 | 0.554 |
|  | Sikasso | 0.07290 | 0.11127 | 0.655 | 0.631 |
|  | SN | - 0.12363 | 0.12848 | - 0.962 | 0.512 |
| *T.v.* prevalence in Senegal | Intercept | 0.1111 | 0.1644 | 0.676 | 0.5689 |
|  | Fleuve gambie | 0.2326 | 0.2014 | 1.155 | 0.3674 |
|  | Mako | 0.8889 | 0.2325 | 3.823 | 0.0621 |
|  | Niokolo | 0.5556 | 0.2325 | 2.389 | 0.1395 |
|  | Tambacounda | 0.2923 | 0.2014 | 1.451 | 0.2838 |
| *T.z.*prevalence in Senegal | Intercept | 0.50000 | 0.03361 | 14.88 | 0.0427 |
|  | Tambacounda | 0.45059 | 0.04116 | - 10.95 | 0.0580 |
| *T.v.* prevalence in Senegal | Intercept | 0.1111 | 0.1644 | 0.676 | 0.5689 |
|  | Fleuve gambie | 0.2326 | 0.2014 | 1.155 | 0.3674 |
|  | Mako | 0.8889 | 0.2325 | 3.823 | 0.0621 |
|  | Niokolo | 0.5556 | 0.2325 | 2.389 | 0.1395 |
|  | Tambacounda | 0.2923 | 0.2014 | 1.451 | 0.2838 |
| T.v. prevalence in Ghana | Intercept | 0.80000 | 0.05279 | 15.155 | 0.00433 |
|  | Bougouhiya | - 0.73750 | 0.07466 | -9.879 | 0.01009 |
|  | Fumbissi | - 0.13333 | 0.07466 | -1.786 | 0.21602 |
|  | Kumpole | - 0.35000 | 0.06465 | -5.413 | 0.03247 |
|  | Mortani | - 0.64375 | 0.07466 | -8.623 | 0.01318 |
|  | Sissili bridge | - 0.60000 | 0.07466 | -8.037 | 0.01513 |
|  | Walewale | - 0.68873 | 0.06465 | -10.653 | 0.00870 |
| T.z. prevalence in Ghana | Intercept | 0.90000 | 0.06721 | 13.392 | 1.07e-05 |
|  | Kumopole | - 0.35000 | 0.09504 | -3.683 | 0.010300 |
|  | Mortani | - 0.65521 | 0.09504 | - 6.894 | 0.000460 |
|  | Fumbissi | - 0.54583 | 0.09504 | - 5.743 | 0.001212 |
|  | Grogro | - 0.71667 | 0.09504 | - 7.540 | 0.000282 |
|  | Kandiaga | - 0.10000 | 0.11640 | 0.859 | 0.423284 |
|  | Psikp | - 0.40000 | 0.11640 | - 3.436 | 0.013863 |
|  | Walewale | -0.73249 | 0.09504 | - 7.707 | 0.000250 |
| T.v. prevalence in Guinea | Intercept | 0.05000 | 0.01511 | 3.309 | 0.187 |
|  | Dekonkore | 0.01250 | 0.02137 | 0.585 | 0.663 |
|  | Karifale | 0.03333 | 0.02137 | 1.560 | 0.363 |
|  | Lemonako | 0.03333 | 0.02137 | 1.560 | 0.363 |
|  | Mini | - 0.01552 | 0.02137 | - 0.726 | 0.600 |
|  | Tinkisso | 0.01624 | 0.01850 | 0.878 | 0.541 |
| Wolbachia prevalence in Guinea | Intercept | 0.08846 | 0.01154 | 7.667 | 0.0826 |
|  | Kifala | 0.03154 | 0.01999 | 1.578 | 0.3596 |
|  | Tinkisso | - 0.03291 | 0.01999 | - 1.647 | 0.3475 |
